# Case treated with triple therapy of lamivudine, interferon-β and prednisolone for acute exacerbation of chronic hepatitis B during pregnancy

**DOI:** 10.1111/j.1872-034X.2012.01077.x

**Published:** 2013-04

**Authors:** Maki Koh, Jun Shinohara, Yasushi Hongo, Tadashi Okazaki, Kimitaka Takitani, Hiroshi Tamai

**Affiliations:** 1 Department of PediatricsHirakata; 2 Department of Gastroenterology and HepatologyHirakata; 3 Department of Obstetrics and Gynecology, Hirakata City HospitalHirakata; 4 Department of Pediatrics, Osaka Medical CollageTakatsuki, Japan

**Keywords:** hepatitis B virus, interferon-β, lamivudine, maternal-infant transmission, pregnancy, steroid

## Abstract

We herein report a case of a pregnant Chinese woman who suffered an acute exacerbation of hepatitis B. The patient's liver enzymes became elevated toward the end of the first trimester. She was treated with lamivudine, interferon (IFN)-β and steroids early in the second trimester. After this treatment regimen was initiated, aminotransferase levels rapidly normalized within 4 weeks. IFN-β and steroids were administrated for 2 weeks in the second trimester, while the administration of lamivudine continued until delivery. The spontaneous delivery of a female baby weighing 2984 g occurred at 37 weeks of gestation. A neonatal examination revealed no congenital anomalies, and fetal growth was found to be within normal reference ranges. The infant received simultaneous active and passive hepatitis B virus immunization within 12 h of delivery and completed the hepatitis B vaccine schedule at 2, 3 and 5 months of age. The infant was successfully prevented from contracting hepatitis B virus. This case suggests that combination therapy with lamivudine, IFN-β and steroids may be safely used during the second trimester to treat acute exacerbations of hepatitis B.

## INTRODUCTION

HEPATITIS B INFECTION is a major medical problem worldwide and causes acute hepatitic failure, chronic hepatitis and hepatocellular carcinoma.^1^,^2^ Approximately one-third of the world's population has a history of hepatitis B virus (HBV) infection. In addition, approximately 350–400 million people are chronically infected with HBV.^2^ HBV infection occurs through vertical or horizontal transmission during infancy and childhood.^3^–^5^ Passive–active immunoprophylaxis with hepatitis B immunoglobulins and the hepatitis B vaccine given at birth is 95% effective at reducing HBV transmission;^6^,^7^ however, vertical transmission of hepatitis B can occur in children despite vaccination. Vaccination breakthrough is associated with high levels of maternal viremia.^8^–^11^ Maternal HBV infection has been reported to be associated with a high incidence of low birthweight and premature birth.^12^ Moreover, acute exacerbations of HBV during pregnancy are associated with high rates of maternal and perinatal mortality.^12^,^13^ Currently, no consensus exists regarding the safety of particular treatments for the acute exacerbations of HBV that occur during pregnancy;^14^ however, some data exist to support the use of lamivudine to reduce viral loads.^15^–^18^ Therefore, the selection of therapy to treat HBV during pregnancy requires careful consideration. In this report, we describe a case of acute exacerbation of HBV during pregnancy that was treated with lamivudine, interferon (IFN)-β and steroids. This therapy regimen successfully improved maternal liver function and reduced the maternal HB viral load. Furthermore, passive and active immunoprophylaxis prevented the perinatal transmission of HBV to the infant.

## CASE REPORT

**Figure 1:**
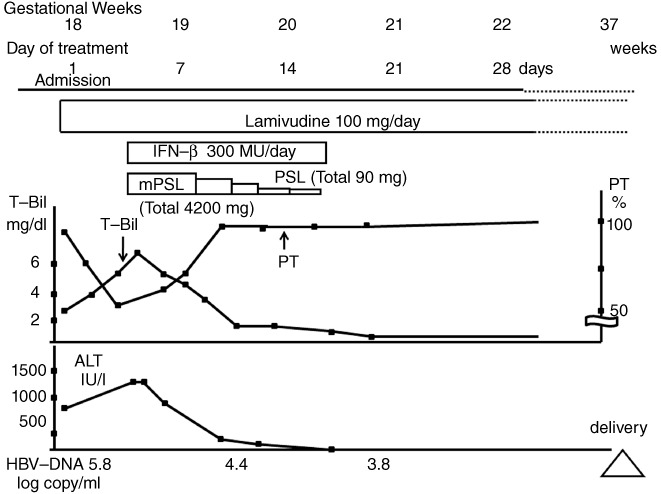
Clinical course of HBV mother with acute exacerbation. ALT, alanine aminotransferase; HBV, hepatitis B virus; IFN-β; interferon-β; mPSL, methylprednisolone; PT, prothrombin time; T-Bil, total bilirubin.

**Figure 2:**
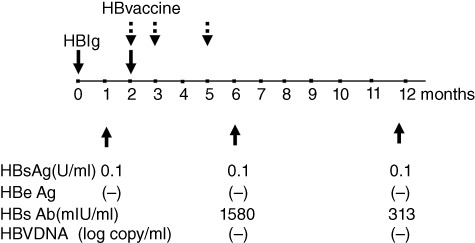
Immunization schedule with HBIg and HBV vaccine, and serological examination for HBV of infant. HBeAg, hepatitis B e-antigen; HBIg; hepatitis B immunoglobulin; HBsAb, hepatitis B surface antibody; HBsAg, hepatitis B surface antigen; HBV, hepatitis B virus.

THIS CASE INVOLVED the second child conceived between a Chinese mother and a Japanese father. The mother was a hepatitis B carrier and her first child had been vaccinated against HBV. The mother was admitted to Hirakata City Hospital at 25 years of age and 15 weeks of gestation. She was found to be negative for hepatitis B e antibody and positive for hepatitis B surface antigen and hepatitis B e-antigen. On admission, her serum aspartate aminotransferase (AST) level measured 915 IU/L, her alanine aminotransferase (ALT) level measured 706 IU/L and her serum HBV DNA showed a high level of 8.2 log copies/mL. The laboratory data indicated an acute exacerbation of hepatitis B. At 18^+6^ weeks of gestation, the patient's ALT level measured 910 IU/L, AST 1426 IU/L and total bilirubin 3.55 mg/dL (Table 1). As the patient was developing liver damage, treatment with lamivudine (100 mg/day) was started (Fig. 1). The Ethical Practices Committee of Hirakata City Hospital approved the study protocol during pregnancy, and the patient gave informed consent. At 19^+2^ weeks of gestation, the patient's aminotransferase levels and hepatic function tests were found to be impaired: ALT, 1250 IU/L; AST, 1892 IU/L; total bilirubin, 5.25 mg/dL; and prothrombin time, 57.2%. In spite of lamivudine administration, aggravation of serum transaminase, total bilirubin and prothrombin time occurred. Thus, the patient was given IFN-β therapy (3 MIU) and methylprednisolone therapy.The patient's aminotransferase levels rapidly normalized within 4 weeks: ALT, 24 IU/L; AST, 28 IU/L; and HBV DNA, 3.8 log copies/mL. IFN-β and steroids (total dose: methylprednisolone 4200 mg and prednisolone 90 mg) were administrated for 2 weeks during the second trimester, and the administration of lamivudine (100 mg/day) was continued until delivery. At 36 weeks of gestation, the patient's serum HBV DNA level had decreased to 2.2 log copies/mL. The spontaneous delivery of a female baby weighing 2984 g occurred at 37^+6^ weeks of gestation. The neonate showed Apgar scores of 9 and 10 at 1 and 5 min, respectively. Fetal growth was found to be within normal reference ranges and a neonatal physical exam revealed no congenital anomalies. Passive immunization with HB immunoglobulins was injected immediately after delivery. The infant completed the HB vaccine schedule at 2, 3 and 5 months of age (Fig. 2). The infant was successfully prevented from contracting HBV infection. The infant received follow-up visits until 1.5 years of age and no adverse effects on the infant's health or development were observed.

**Table 1:** Laboratory findings of the mother with acute exacerbation of hepatitis B virus

Laboratory test	Levels	Reference range
White blood cells (10^3^/µL)	7.6	(4.7–8.7)
Red blood cells (10^6^/µL)	3.91	(3.70–4.90)
Hemoglobin (g/dL)	12.4	(12.0–16.0)
Platelets (10^3^/µL)	195	(150–400)
Aspartate aminotransferase (IU/L)	1,426	(8–38)
Alanine aminotransferase (IU/L)	910	(4–44)
Lactate dehydrogenase (IU/L)	475	(106–211)
γ-Glutamyl transpeptidase (IU/L)	51	(16–73)
Cholinesterase (IU/L)	120	(185–431)
Total protein (g/dL)	5.6	(6.7–8.3)
Albumin (g/dL)	2.8	(3.8–5.3)
Ammonia (µg/dL)	24	(12–66)
Total bilirubin (mg/dL)	3.55	(0.2–1.2)
Direct bilirubin (mg/dL)	2.46	(0.00–0.4)
Total cholesterol (mg/dL)	158	(130–219)
Blood urea nitrogen (mg/dL)	5.3	(7–18)
Creatinine (mg/dL)	0.42	(0.4–0.8)
Na (mEq/L)	138	(135–145)
K (mEq/L)	3.8	(3.5–5.0)
Prothrombin time (%)	97.9	(60–100)
Prothrombin time – International Normalized Ratio	1.02	(0.95–1.35)

## DISCUSSION

IN THIS CASE, sequential therapy with lamivudine, IFN-β and steroids was administrated during the second trimester to treat an acute exacerbation of HB. This therapy reduced the maternal HBV DNA viral load without showing any negative effects on the fetus. Intensive treatment of HBV after second trimester exposure has been proven to be well-tolerated. Moreover, comprehensive immunoprophylaxis for infants can prevent the transmission of HBV. However, the balance of the potential benefits and risks of treatment of HB infection during pregnancy for both the mother and the infant should be considered. Currently, data exist to support maternal treatment with lamivudine in order to reduce HBV DNA levels.^15^–^18^ Lamivudine, an oral cytosine nucleoside analog, has been widely used in pregnant women with HIV because it is efficacious and well-tolerated.^19^–^21^ Furthermore, lamivudine therapy has been proven to be effective in HBV-infected pregnant women and its use in pregnancy is likely to be advisable.^22^,^23^ In the present case, lamivudine therapy alone did not improve maternal liver function. An acute exacerbation of HBV during pregnancy is a critical complication that is associated with maternal and prenatal mortality.^12^,^13^ After administrating lamivudine therapy, we decided to initiate treatment with IFN-β and steroids (methylprednisolone followed by prednisolone) in order to prevent the development of hepatitis.

IFN-β may not be considered safe for use during pregnancy due to its anti-proliferative effects.^24^,^25^ The US Food and Drug Administration classifies IFN as a pregnancy category C drug (animal reproduction studies have shown adverse effects of IFN on fetuses, and there exist no well-controlled studies in humans).^26^ IFN-β is an immunomodulator that is used to prevent relapse and progression in patients with multiple sclerosis (MS).^27^ Several clinical studies have proven that *in utero* exposure to IFN-β during pregnancy occurs in patients with MS; however, results regarding the effects of IFN-β on pregnant mothers and newborns have been conflicting.^28^–^32^ Boskovic and colleagues reported that exposure to IFN-β in the first trimester increases the risk for miscarriages, stillbirth and low infant birthweight.^28^ A study by Amato *et al*. revealed that IFN-β exposure does not increase the risk of spontaneous abortion and is not associated with any significant fetal complications, malformations or developmental abnormalities; however, this study found that IFN-β exposure is associated with low birthweight and shorter length in newborns.^29^ Other recent studies on *in utero* exposure to IFN-β in patients with MS have shown that IFN-β does not affect maternal or infant outcomes.^31^,^32^ However, no previous studies have so far investigated the *in utero* exposure in patients with HBV infection. In the present case, the patient was treated with IFN-β for 2 weeks in the second trimester. This report suggests that the use of IFN to treat HBV infection is beneficial, not only for improving liver function, but also for reducing HBV viral loads.

The administration of steroid therapy during pregnancy may potentially influence the incidence of intrauterine growth retardation.^33^ For steroids, the risk to the fetus is correlated with the transplacental passage rate.^34^ Because prednisolone is inactivated by 11 β-hydroxysteroid dehydrogenase in the placenta, the effects of steroid use on the fetus during pregnancy may be small.^34^ On the other hand, methylprednisolone passes through the placental barrier, and fetal levels of methylprednisolone are typically approximately one-third of maternal levels.^34^ Therefore, we were aware that the potential benefits of these agents outweighed the potential risks for both the mother and the fetus, and these agents were administrated with discretion. In this case, combination therapy with lamivudine, IFN-β and steroids administrated during the second trimester has achieved sufficient reductions in maternal HBV DNA levels.^35^

Regarding the prevention of perinatal HBV transmission, some studies have reported beneficial effects from the use of HBV vaccines and hepatitis B immunoglobulins in infants. Passive–active immunoprophylaxis in infants in combination with maternal therapy to reduce HBV DNA levels can prevent maternal–infant HBV transmission. This case report demonstrates the potential efficacy and safety of treatment with lamivudine, IFN-β and steroids during the second trimester. However, there is the limitation of this being a case report, so further clinical studies are needed to evaluate the efficacy and safety of maternal therapy with these agents to treat acute exacerbations of HBV during pregnancy.
